# Cone beam CT-based dose accumulation and analysis of delivered dose to the dominant intraprostatic lesion in primary radiotherapy of prostate cancer

**DOI:** 10.1186/s13014-021-01933-z

**Published:** 2021-10-26

**Authors:** Jörg Tamihardja, Sinan Cirsi, Patrick Kessler, Gary Razinskas, Florian Exner, Anne Richter, Bülent Polat, Michael Flentje

**Affiliations:** Department of Radiation Oncology, University of Wuerzburg, Josef-Schneider-Str. 11, 97080 Würzburg, Germany

**Keywords:** Adaptive radiotherapy, Deformable image registration, Dominant intraprostatic lesion, Dose accumulation, Prostate cancer, Prostate Imaging Reporting and Data System

## Abstract

**Background:**

Evaluation of delivered dose to the dominant intraprostatic lesion (DIL) for moderately hypofractionated radiotherapy of prostate cancer by cone beam computed tomography (CBCT)-based dose accumulation and target coverage analysis.

**Methods:**

Twenty-three patients with localized prostate cancer treated with moderately hypofractionated prostate radiotherapy with simultaneous integrated boost (SIB) between December 2016 and February 2020 were retrospectively analyzed. Included patients were required to have an identifiable DIL on bi-parametric planning magnetic resonance imaging (MRI). After import into the RayStation treatment planning system and application of a step-wise density override, the fractional doses were computed on each CBCT and were consecutively mapped onto the planning CT via a deformation vector field derived from deformable image registration. Fractional doses were accumulated for all CBCTs and interpolated for missing CBCTs, resulting in the delivered dose for PTV_DIL_, PTV_Boost_, PTV, and the organs at risk. The location of the index lesions was recorded according to the sector map of the Prostate Imaging Reporting and Data System (PIRADS) Version 2.1. Target coverage of the index lesions was evaluated and stratified for location.

**Results:**

In total, 338 CBCTs were available for analysis. Dose accumulation target coverage of PTV_DIL_, PTV_Boost_, and PTV was excellent and no cases of underdosage in D_Mean_, D_95%_, D_02%_, and D_98%_ could be detected. Delivered rectum D_Mean_ did not significantly differ from the planned dose. Bladder mean D_Mean_ was higher than planned with 19.4 ± 7.4 Gy versus 18.8 ± 7.5 Gy, p < 0.001. The penile bulb showed a decreased delivered mean D_Mean_ with 29.1 ± 14.0 Gy versus 29.8 ± 14.4 Gy, p < 0.001. Dorsal DILs, defined as DILs in the posterior medial peripheral zone of the prostate, showed a significantly lower delivered dose with a mean D_Mean_ difference of 2.2 Gy (95% CI 1.3–3.1 Gy, p < 0.001) compared to ventral lesions.

**Conclusions:**

CBCT-based dose accumulation showed an adequate delivered dose to the dominant intraprostatic lesion and organs at risk within planning limits. Cautious evaluation of the target coverage for index lesions adjacent to the rectum is warranted to avoid underdosage.

## Background

Radiotherapy is an established treatment option for one of the most common cancer types in men: prostate cancer [1]. While whole-gland radiotherapy is the standard treatment modality for prostate cancer, focal therapy may result in decreased toxicity while maintaining high tumor control. For the prostate, the relevant target structure for focal therapy is the dominant intraprostatic lesion (DIL) or index lesion as local recurrence often occurs at the site of the initial primary tumor which influences prognosis and disease progression [2–5]. The implementation of focal ablative micro boosts to the DIL, as investigated in the FLAME trial, depends on accurate planning and treatment delivery [6]. To achieve the goal of precise and highly conformal dose distributions, MRI- and CBCT-based positioning monitoring methods are commonly implemented [7]. Nevertheless, planned and delivered doses may differ due to motion and daily variations in rectum and bladder filling states. Especially for focal targeted therapy of the index lesion with tight margins, adequate dose delivery is of utmost importance to avoid target miss and underdosage. Dose accumulation strategies may therefore guide the clinical decision to adapt the current radiotherapy plan during treatment. In this study, a CBCT-based dose accumulation for the dominant intraprostatic lesion in moderately hypofractionated volumetric arc radiotherapy (VMAT) with SIB was performed as a step towards dose-guided adaptive prostate radiotherapy.

## Methods

### Patient characteristics and treatment planning

**Fig. 1:**
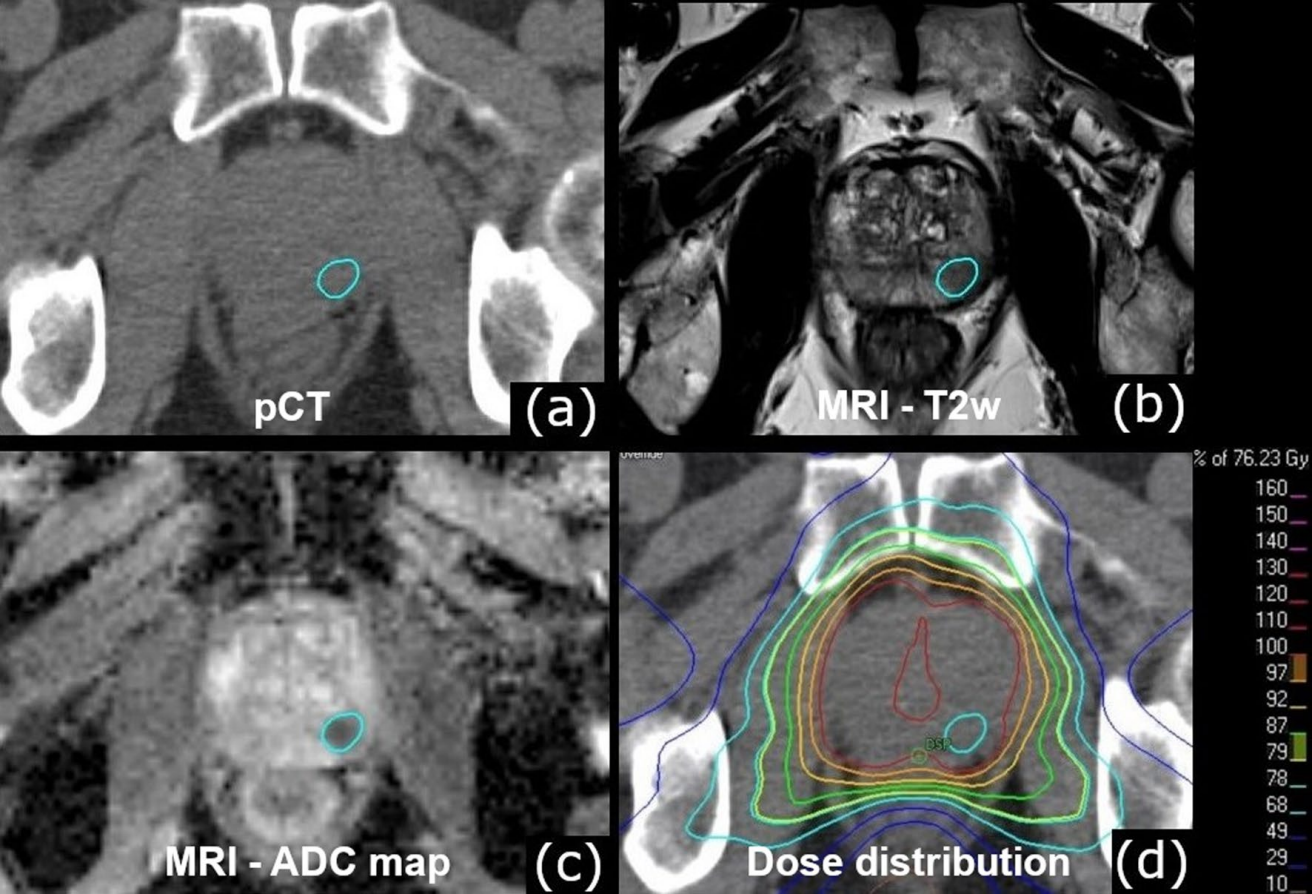
Dominant intraprostatic lesion contouring. Shown is the index lesion in the peripheral zone on the **a** planning CT, **b** native T2-weighted image, **c** apparent diffusion coefficient map, **d** planned dose distribution. Abbreviations: *pCT* planning CT, *MRI* Magnetic resonance imaging, *T2w* T2-weighted, *ADC map* apparent diffusion coefficient map

Twenty-three patients with histologically proven low- to high-risk prostate cancer and identifiable DIL in the pre-radiotherapy planning MRI underwent image-guided radiotherapy (IGRT) with CBCT-guidance at an Elekta Synergy Agility linear accelerator (Elekta AB, Stockholm, Sweden) from December 2016 to February 2020. All patients received computed tomography (CT) and bi-parametric MRI scans for radiotherapy planning in a supine position. For the CT scan and the radiotherapy appliance, the patients were instructed to have a full bladder and an empty rectum. Planning MRI protocol consisted of a T2-weighted turbo spin-echo sequence and diffusion-weighted imaging [8]. Radiotherapy was delivered with image-guided VMAT in 33 fractions with simultaneous integrated boost. Regarding the patient set-up workflow, a bone to bone match restricted to the target area was applied first. Afterwards, a consecutive manual soft-tissue matching to the target volumes was performed. Three degrees of freedom shifts were applied without rotational compensation. CBCT scans were performed on each of the first five fractions and once every three fractions afterwards. Contouring, dose prescription, and treatment planning have been reported in detail in earlier publications [8, 9]. In short, prostate radiotherapy was planned with two dose levels of 1.82 Gy for the low dose PTV and 2.31 Gy for the high dose PTV_Boost_ per fraction, resulting in a prescribed PTV dose of 60.06 Gy (D_95%_) and a PTV_Boost_ mean dose of 76.23 Gy. A clinical target volume (CTV_P−SV_) was generated consisting of the prostate and only the base of the seminal vesicles, whereas the CTV_P+SV_ included the prostate and the whole seminal vesicles. PTV_Boost_ was defined by a 5 mm margin around CTV_P−SV_ with avoidance of the rectum. The PTV was created by a 10 mm margin around CTV_P+SV_ in all but the dorsal direction, where a 7 mm margin was used. The planning MRI was rigidly co-registered to the planning CT with soft-tissue matching in the target area. Contouring of the clinical target volumes took place on the fused MRI and planning CT without additional contour transferring. PTV_DIL_ was defined as the DIL without margin and contoured by an experienced radiation oncologist according to the co-registered planning MRI sequences. The contouring of PTV_DIL_ is illustrated in Fig. 1. Pinnacle^3^ version 16.2 (Philips Radiation Oncology Systems, Fitchburg, WI, USA) was used as treatment planning system (TPS). RayStation version 6.99 (RaySearch Laboratories AB, Stockholm, Sweden) was used for analysis.

### Dose accumulation

**Fig. 2:**
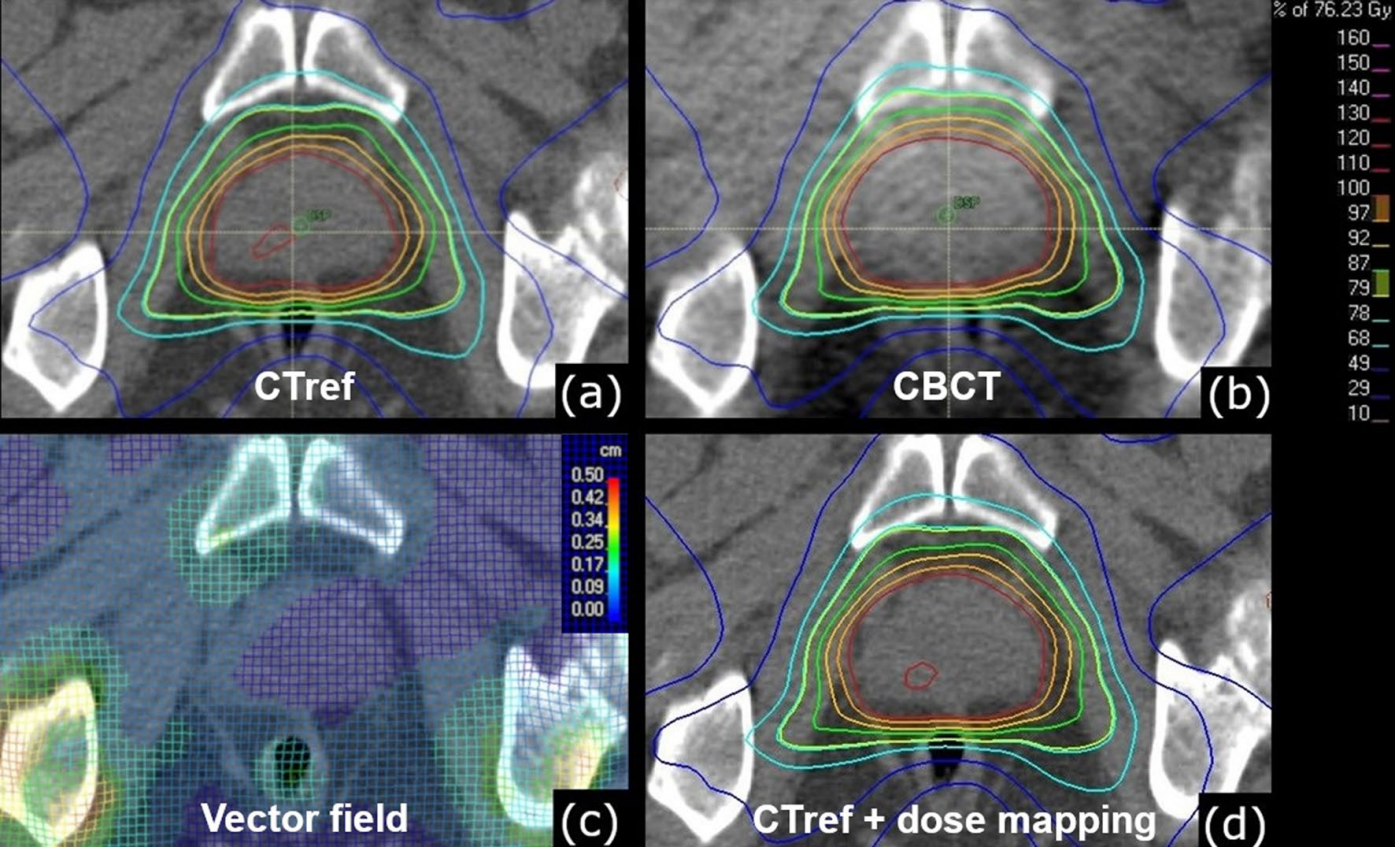
Illustration of the dose computation workflow. After import into the RayStation TPS and application of a density override, the dose is computed on the density overridden pCT (CT_ref_) (**a**) and on the density overridden CBCT (**b**). The dose computed on each CBCT is consecutively mapped via a deformation vector field (**c**), derived from deformable image registration, onto CT_ref_ (**d**). The vector field is color-coded from blue (0.0 cm deformation) to red (0.5 cm deformation). Abbreviation: *CT*_*ref*_ reference CT

The workflow for generating a CBCT-based dose calculation and dose accumulation is described in the following section and illustrated in Fig. 2:

1. *Import into the RayStation TPS:* The original patient’s plan, which was generated with the Pinnacle^3^ TPS, as well as all CBCTs were transferred to the RayStation TPS. The Raystation TPS was chosen as primary TPS for this study because of RayStation`s deformable image registration algorithm, which was proven to be accurate and reliable in the literature [10, 11]. The target volumes, the organ at risk (OAR) contours and couch shift data from the kilovoltage X-ray volume imaging system (Elekta AB, Stockholm, Sweden) were imported into RayStation. The extent of the couch shift in the three main axes (left–right, superior–inferior, anterior–posterior) was analyzed.
2. *Density override of CBCT/planning CT:* A density override for each CBCT and planning CT (pCT) was performed within the RayStation TPS to reduce the influence of Houndsfield unit (HU) variability on the dose calculation. In the RayStation TPS, HU ranges of all CBCTs and planning CTs were used to select areas with the respective HU range before density override. Thereby, a density table with five density levels (air, lung, adipose tissue, cartilage/bone, and higher density for prosthesis) was created for all CBCTs and pCTs, resulting in a stepwise HU-to-density conversion curve [12]. The corrected pCT served as reference CT (CT_ref_). The density override was applied to both the CBCTs as well as the pCTs. Therefore, the dose calculation error by density overriding is found in both calculated delivered and calculated planned dose in the RayStation TPS, which allows the comparison of both. The density overridden CBCTs and pCTs were utilized in the deformable image registration (DIR) process as described in the following sections.
3. *Dose calculation on CT*_*ref*_*:* The patient`s plan was recalculated on the CT_ref_ with overrridden step-wise density in the RayStation TPS and served as the reference plan.
4. *Rigid registration and dose calculation on CBCT:* Next, each CBCT was rigidly co-registered to CT_ref_ according to the couch shift information from the integrated XVI system. This ensured that the actual treatment position of the patient with reference to the isocenter was taken into account. Afterwards, the dose distribution of the patient’s plan was calculated on each CBCT.
5. *Deformable image registration (DIR):* A DIR of the CBCT on CT_ref_ was performed which generated a deformation vector field by the appliance of the RayStation ANAtomically CONstrained Deformation Algorithm (ANACONDA)(10, 11). The ANACONDA algorithm measures image similarity by the appliance of a correlation coefficient. The DIR algorithm as described here offers a hybrid registration solution, combining both geometric and intensity information [10]. Because density overriding results in areas with homogeneous density, the algorithm mostly acts on organ boundaries. Target volumes did not change during treatment and DIR was not challenged by changing target volumes during treatment.
6. *Dose mapping:* The CBCT dose distribution was remapped back on CT_ref_ by utilizing the obtained deformation vector field. This resulted in the CBCT dose distribution on CT_ref_, calculated out of the original plan on the RayStation TPS.
7. *Dose accumulation:* The CBCT dose distribution on the CT_ref_, calculated out of the original plan on the RayStation TPS as described above, was used to calculate the fractional dose for each CBCT. In case that no CBCT was performed on a given day, the previous CBCT was selected for fractional dose calculation instead, and it was assumed that organ deformation stayed similar. By summation of all fractional doses a dose accumulation for PTV_DIL_, PTV_Boost_, PTV, and the organs at risk rectum, bladder, femoral heads, and penile bulb was achieved.

### Dominant intraprostatic lesion location and accumulated dose

**Fig. 3:**
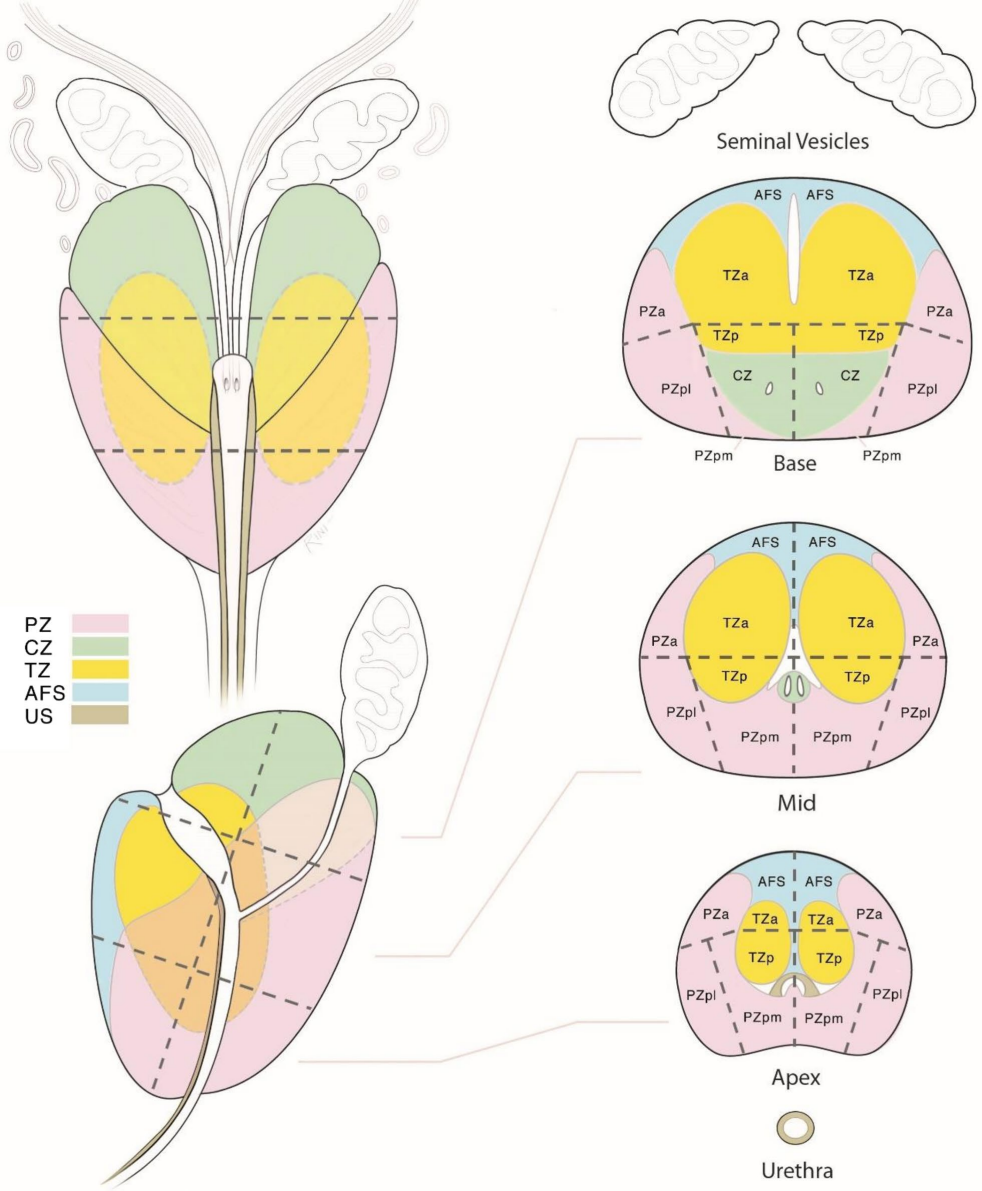
PIRADS 2.1 sector map [14]. Permission for publication obtained from American College of Radiology Committee on PI-RADS®, licensed under Creative Commons Attribution-NoDerivatives 4.0 International (CC BY-ND 4.0). Abbreviations: *AFS* anterior fibromuscular stroma, *CZ* central zone, *TZa/p* transition zone, anterior/posterior, *PZa/pm/pl* peripheral zone, anterior/posterior medial/posterior lateral, *US* urethra

The location of all index lesions was reviewed and classified according to the sector map of the American College of Radiology Prostate Imaging Reporting and Data System (PIRADS) Version 2.1, which is illustrated in Fig. 3 [13]. The accumulated dose for PTV_DIL_ was analyzed for differences between ventral and dorsal index lesions. Dorsal location was defined as location inside the posterior medial peripheral zone (PZpm) of the prostate. Ventral location was defined as all other locations inside the prostate.

### Statistics

Matched-pairs t-tests were applied for the comparison of planned and accumulated doses for normally distributed parameters according to the Shapiro–Wilk test. In the case of non-normally distributed parameters, Wilcoxon matched-pairs signed-rank tests were applied instead. An unpaired t-test was used for the evaluation of differences in the accumulated dose according to index lesion location. Statistical significance was declared in the case of a two-sided p < 0.05. Statistical analysis was conducted using IBM SPSS v.26.0 (IBM Corp., Armonk, NY, USA). Descriptive quantitative values are expressed as mean ± standard deviation or as median with the corresponding range as appropriate.

## Results

### Patient characteristics

The median patient age was 71 years (57–83 years) with a median Karnofsky Performance Status of 100% (70–100%). The mean initial prostate-specific antigen (PSA) value reached 9.2 ± 5.9 ng/mL and 5/13/5 patients had a Gleason score of 6/7/8–10, respectively. There were 5 cases of low-risk, 11 cases of intermediate-risk, and 7 cases of high-risk prostate cancer after the D`Amico risk classification [15]. The patient characteristics are summarized in Table 1.

**Table 1:** Patient characteristics

Characteristics	
Median age in years (range)	71 (57–83)
Median KPS in % (range)	100 (70–100)
Median iPSA in ng/mL (range)	7.9 (3.3–25.4)
Median DIL volume in cm^3^ (range)	1.4 (0.4–6.6)
Gleason score	
6	5 (21.7%)
7	13 (56.5%)
8–10	5 (21.7%)
TNM stage	
cT1a-c	21 (91.3%)
cT2a-c	2 (8.7%)
D’Amico risk group	
Low-risk	5 (21.7%)
Intermediate-risk	11 (47.8%)
High-risk	7 (30.4%)
Index lesion sector distribution	
Anterior fibromuscular stroma	8/23 (34.8%)
Anterior TZ	6/23 (26.1%)
Posterior TZ	5/23 (21.7%%)
Anterior PZ	2/23 (8.7%)
Posterior medial PZ	8/23 (34.8%)
Posterior lateral PZ	11/23 (47.8%)
Central zone	0/23 (0.0%)

*KPS* Karnofsky Performance Status, *iPSA* initial prostate-specific antigen, *DIL* dominant intraprostatic lesion, *TZ* transition zone, *PZ* peripheral zone

### X-ray volume imaging couch shift analysis

Overall, 338 CBCTs were acquired with mean 14.7 ± 1.9 CBCTs per patient. The mean (± SD) couch shift in the three main axes were: 0.1 ± 2.9 mm (left–right), − 0.4 ± 2.7 mm (superior-inferior), − 0.0 ± 2.8 mm (anterior–posterior). Median shift for left–right was 0 cm (− 7 to 8 mm), median shift for superior-inferior was 0 cm (− 9 to 7 mm), median shift for anterior–posterior was 0 cm (− 9 to 8 mm). Regarding shift distribution, 95.8% of all shifts in the three axes were within 5 mm and 64% were within 2 mm. 0.8% of all shifts were greater than 7 mm.

### Dose accumulation

Target coverage of PTV_Boost_ and PTV was excellent and no cases of underdosage in the accumulated doses for PTV_DIL_, PTV_Boost_, and PTV in D_Mean_, D_95%_, D_02%_, and D_98%_ could be detected. For the organ at risk rectum, mean D_Mean_ was not significantly different between dose accumulation and initial treatment plans. Delivered bladder mean D_Mean_ was higher than planned with 19.4 ± 7.4 Gy versus 18.8 ± 7.5 Gy, p < 0.001. The penile bulb showed a slightly decreased delivered mean D_Mean_ with 29.1 ± 14.0 Gy versus 29.8 ± 14.4 Gy, p < 0.001. The target coverage and organ at risk parameters are summarized in Table 2.

**Table 2:** Target volume coverage and dose to organs at risk

	Dose accumulation	Planned dose	p
PTV_DIL_			
D_98%_ (Gy)	76.2 ± 1.6	75.6 ± 1.4	< 0.001*
D_02%_ (Gy)	79.6 ± 1.5	79.1 ± 1.2	< 0.001*
D_95%_ (Gy)	76.5 ± 1.5	75.9 ± 1.3	< 0.001*
D_Mean_ (Gy)	78.0 ± 1.4	77.4 ± 1.2	< 0.001*
PTV_Boost_			
D_98%_ (Gy)	71.1 ± 0.8	71.2 ± 0.7	0.750*
D_02%_ (Gy)	79.9 ± 0.8	79.4 ± 0.4	0.001
D_95%_ (Gy)	72.6 ± 0.7	72.5 ± 0.5	0.288
D_Mean_ (Gy)	76.5 ± 0.6	76.1 ± 0.2	0.001*
PTV			
D_98%_ (Gy)	57.5 ± 1.1	57.9 ± 0.7	0.129*
D_02%_ (Gy)	79.5 ± 0.8	79.0 ± 0.4	0.001
D_95%_ (Gy)	59.7 ± 0.8	59.6 ± 0.7	0.200
D_Mean_ (Gy)	70.8 ± 1.3	70.4 ± 0.9	0.003
Rectum			
D_Mean_ (Gy)	23.4 ± 6.9	23.6 ± 6.6	0.353
Bladder			
D_Mean_ (Gy)	19.4 ± 7.4	18.8 ± 7.5	< 0.001*
Femoral head left			
D_Mean_ (Gy)	16.1 ± 6.6	15.5 ± 6.4	0.001
Femoral head right			
D_Mean_ (Gy)	15.7 ± 5.0	14.8 ± 4.8	< 0.001*
Penile bulb			
D_Mean_ (Gy)	29.1 ± 14.0	29.8 ± 14.4	< 0.001

Comparison between planned and accumulated dose for the target volumes and organs at risk. Statistical significance was tested by matched-pairs t-tests in case of normally distributed parameters. In case of non-normally distributed parameters Wilcoxon matched-pairs signed rank tests (*) were applied instead. Statistical significance was declared in case of a two-sided p < 0.05. Stated values indicate mean ± standard deviation

### Dominant intraprostatic lesion

**Fig. 4:**
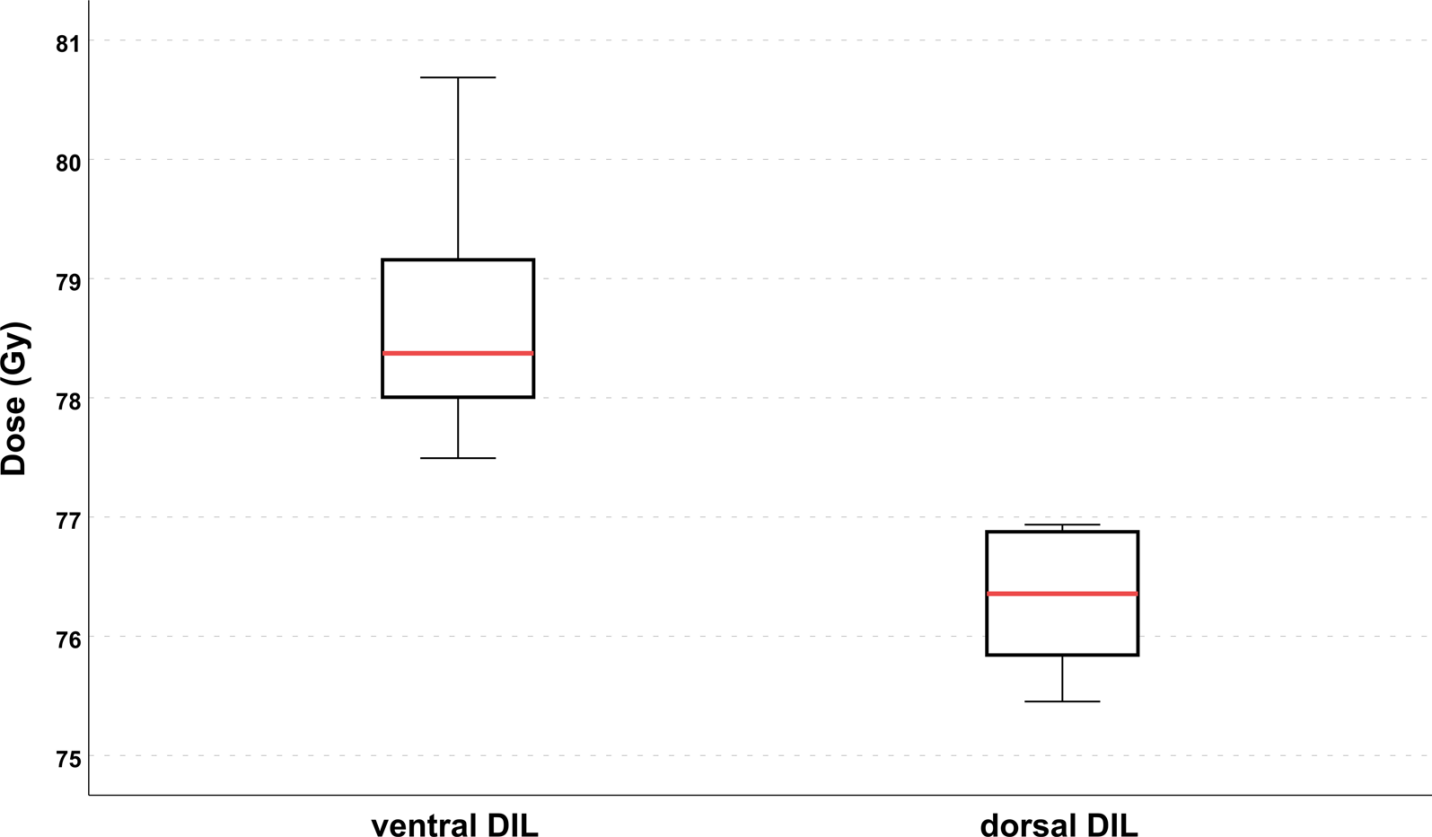
Effect of DIL location on the applied dose. Shown is the boxplot of the delivered mean dose in Gy for PTV_DIL_ for ventral and dorsal index lesions. Dorsal index lesions, defined as index lesions in the posterior medial peripheral zone of the prostate, showed a significantly lower mean D_Mean_ (p < 0.001)

The mean volume of the PTV_DIL_ was 2.2 ± 2.1 cm^3^. 23 index lesions were identified. The sector distribution is summarized in Table 1. Eight out of 23 index lesions (34.8%) were located dorsally adjacent to the OAR rectum in the posterior medial peripheral zone (PZpm) according to the classification of PIRADS 2.1. Location of the index lesion in the PZpm had a statistically significant impact on the applied dose: D_Mean_ of the index lesions in the PZpm was significantly lower (76.5 ± 0.4 Gy, n = 8) than for index lesions in the rest of the prostate (78.7 ± 0.3 Gy, n = 15), p < 0.001, t-value = 4.921 (21 df). The mean D_Mean_ difference was 2.2 Gy (95% CI 1.3–3.1 Gy). The effect strength according to Cohen was 0.73, which represents a strong effect (Fig. 4).

## Discussion

Dose-guided adaptive radiotherapy for external beam radiotherapy is increasingly used to adjust to anatomy changes and to avoid underdosage of the target volumes due to motion and organ deformation [16]. Dose accumulation and the CBCT-derived fractional daily dose may offer a feedback mechanism to trigger the adaption of radiotherapy. For focal prostate radiotherapy, the critical target structure is the index lesion and to our knowledge, this is the first study to investigate the dose coverage of the DIL in prostate cancer by CBCT-based dose accumulation.

Dose accumulation for prostate radiotherapy in general has been evaluated before: In a recent analysis, Ong et al. presented a workflow for dose accumulation of prostate and pelvic lymph node radiotherapy with two isocenters. Due to different coordinate systems in the utilized MIM v.6.9 (MIMVista Corp., Cleveland OH) and MosaiQ (Elekta AB, Stockholm, Sweden) software Ong et al. had to perform phantom measurements for couch shift commissioning which allowed the import of MosaiQ extracted, correctly aligned shift information into the contouring software [17]. In our current analysis, the RayStation TPS allowed the direct import and usage of couch shift data and therefore phantom measurements were omitted. The analysis of shift distribution demonstrated significantly, but non-excessive couch shifts. Over 95% of all shifts were adequately covered within 5 mm and therefore within the high dose PTV (PTV_Boost_). 4.2% and 0.8% of all shifts exceeded 5 mm, respective 7 mm. This underlines the importance of inter-fractional dose accumulation and also confirms the utilized institutional margin concept. The delivered dose demonstrated excellent target coverage of the target volumes and the dominant intraprostatic lesion. No cases of underdosage for the PTV, PTV_Boost_ as well as PTV_DIL_ could be detected.

The delivered mean dose was significantly lower for dorsal DILs which may be attributable to the dose fall-off at the edges of PTV_Boost_ as a steep dose gradient for sparing the OAR rectum was utilized. This is in line with an earlier work that showed decreased planned dose for dorsal index lesions in prostate radiotherapy [8]. Despite no margin between the rectum and PTV_Boost_, a long-term analysis of the utilized regime of moderately hypofractionated prostate radiotherapy with the institutional target volume and margin concept showed excellent biochemical relapse-free survival and low rates of gastrointestinal toxicity [9]. In the current work, dose accumulation showed excellent agreement between planned and delivered rectum mean D_Mean._ The delivered dose to the bladder was higher than planned which emphasizes the importance of strict adherence to bladder filling protocols to avoid genitourinary toxicity. In a future analysis, the presented workflow should be explored further for the correlation between delivered dose and clinical toxicity as delivered dose has been prognostic for unwanted sequelae in the literature [18]. In case of severe CBCT artifacts in the target area our approach may not be possible. CBCT artifacts in the target area which need to be mitigated for our approach are especially scatter artifacts, extinction artifacts, and beam hardening artifacts [19]. Scatter artifacts reduce soft-tissue contrast and will affect the density values of the tissues. For highly absorbing material in the target area, the signal recorded in the detector behind the material may be close to zero, resulting in extinction artifacts. The absorption of lower energetic rays, for example by titanium in hip total endoprosthesis, will cause massive beam hardening artifacts for pelvic CBCTs [19]. In our study cohort, we did not observe severe CBCT artifacts. A quality assurance procedure for the presented workflow would have to incorporate the verification of the density override and DIR algorithm accuracy. A possible solution would be to use another dedicated software or treatment planning system to compare the results with the RayStation generated ones. To quantify the differences, a metric would have to be developed which would be used for defining action thresholds. The development of a quality assurance procedure for the dose accumulation workflow was beyond the scope of this study.

Our study has limitations: The accuracy of CBCT derived dose is determined by relative electron density and therefore correlated with HU. CBCT quality has a major influence on density calculation and therefore may have introduced uncertainty in the dose accumulation analysis. As detailed in the methods section, although a density assignment as correction strategy was applied, the calculated absolute dose values suffer from uncertainties due to step-wise instead of continuous conversion curves [12]. Dunlop et al. showed an absolute mean dose error of 0.7% for pelvic CBCT-based step-wise dose calculation in case of an anterior–posterior distance < 25 cm [12]. In our study, we estimated the error of our approach by comparing the mean dose difference between pCT and CT_ref_, which amounted to 0.7% for PTV_Boost_. DIR quality also influences the robustness of the dose accumulation, but the ANACONDA DIR algorithm was shown to have high accuracy in the literature [11]. Another limitation is the lack of daily CBCTs according to the institutional imaging protocol which foresees daily CBCT only in the beginning when the patient is not accustomed to the organ preparation protocols. Later on, when the patient is trained, organ filling states remain mostly stable in clinical practice. We recently published our data for definitive prostate radiotherapy using this IGRT protocol showing no detrimental effect on biochemical relapse-free survival [9]. As strict adherence to organ preparation protocols is still mandatory, selecting the closest temporal CBCT in case of missing CBCTs is expected to have no excessive impact on the dose accumulation accuracy. Nonetheless, daily instead of non-daily CBCT may further improve the dose accumulation workflow and will be explored in sequential studies [20, 21]. Our data is also limited by the absence of intra-fractional motion analysis and correction, which was not available for the analyzed patient cohort. In particular for stereotactic radiotherapy, but as well as moderately fractionated radiotherapy, target motion during delivery can be significant and influence delivered dose. Using VMAT instead of step & shoot IMRT, the treatment time per fraction is reduced, which translates to a decreased risk of intra-fractional organ changes [22]. As next step, incorporating intra-fractional prostate motion into the dose accumulation workflow will be investigated. Lastly, the retrospective character and small patient sample of the current analysis warrants confirmation in a bigger patient cohort.

To look further, artificial intelligence may improve CBCT correction and dose calculation as has been highlighted recently: Maspero et al. trained a single neural network to generate synthetic CTs (sCT) out of CBCT for head-and-neck, lung, and breast cancer, enabling sCT-based dose calculation with under 0.5% mean dose difference to re-planning CTs which is lower than the estimated error of 0.7% in our study, although a direct comparison was outside the scope of our analysis [23]. Deep learning-based dose calculation, together with automatic segmentation, may represent crucial steps on the way to fully automatic dose-guided adaptive radiotherapy in general and especially for focal boost radiotherapy with tight margins. Regarding dose-escalation to the index lesion, the FLAME trial recently showed that a high dose focal boost improves biochemical disease-free survival in intermediate- and high-risk localized prostate cancer without additional toxicity [6]. The correlation between delivered dose to the index lesion, derived by dose accumulation, and clinical outcome remains an interesting research topic.

## Conclusions

A workflow for the calculation of the delivered dose to the dominant intraprostatic lesion using CBCT-based dose accumulation on the RayStation TPS was established. The delivered dose to the dominant intraprostatic lesion and organs at risk was adequate and well within planning limits. Dorsal index lesions demonstrated a lower applied mean dose than ventral lesions, but no cases of underdosage were observable.

## Data Availability

The datasets used and/or analyzed during the current study are available from the corresponding author on reasonable request.

## Abbreviations

ADC map: Apparent diffusion coefficient map
AFS: Anterior fibromuscular stroma
CBCT: Cone beam computed tomography
CI: Confidence interval
CT: Computed tomography
CT_ref_: Reference computed tomography
CTV: Clinical target volume
CZ: Central zone
Df: Degrees of freedom
D_Mean_: Mean dose
DIL: Dominant intraprostatic lesion
DIR: Deformable image registration
D_X%_: Dose to X% of the target volume
HU: Hounsfield units
IGRT: Image-guided radiotherapy
KPS: Karnofsky Performance Status
MRI: Magnetic resonance imaging
OAR: Organs at risk
pCT: Planning CT
PIRADS: Prostate Imaging Reporting and Data System
PSA: Prostate-specific antigen
PTV: Planning target volume
PZa/pm/pl: Peripheral zone, anterior/posterior medial/posterior lateral
sCT: Synthetic CT
SIB: Simultaneous integrated boost
T2w: T2-weighted
TPS: Treatment planning system
TZa/p: Transition zone, anterior/posterior
VMAT: Volumetric arc radiotherapy
US: Urethra
XVI: X-ray volume imaging
